# Promising Role of Emodin as Therapeutics to Against Viral Infections

**DOI:** 10.3389/fphar.2022.902626

**Published:** 2022-05-04

**Authors:** Qingqing Shao, Tong Liu, Wenjia Wang, Tianli Liu, Ximing Jin, Zhuo Chen

**Affiliations:** ^1^ Institute of Integrated Traditional Chinese and Western Medicine, Tongji Hospital, Tongji Medical College, Huazhong University of Science and Technology, Wuhan, China; ^2^ Department of Integrated Traditional Chinese and Western Medicine, Tongji Hospital, Tongji Medical College, Huazhong University of Science and Technology, Wuhan, China

**Keywords:** emodin, virus infection, HSV-2, HCMV (human cytomegalovirus), COVID-19

## Abstract

Emodin is an anthraquinone derivative that is widely present in natural plants and has a wide spectrum of pharmacological effects, such as antibacterial, anti-inflammatory, anti-fibrotic and anticancer and so on. Through reviewing studies on antiviral effect of emodin in the past decades, we found that emodin exhibits ability of inhibiting the infection and replication of more than 10 viruses *in vitro* and *in vivo*, including herpes simplex virus type 1 (HSV-1) and type 2 (HSV-2), human cytomegalovirus (HCMV), Epstein-Barr virus (EBV), coxsackievirus B (CVB), hepatitis B virus (HBV), influenza A virus (IAV), SARS-CoV, viral haemorrhagic septicaemia rhabdovirus (VHSV), enterovirus 71 (EV71), dengue virus serotype 2 (DENV-2) and Zika virus (ZIKV). Therefore, this review aims to summarize the antiviral effect of emodin, in order to provide reference and hopes to support the further investigations.

## Introduction

Emodin (1,3,8-trihydroxy-6-methylanthraquinone, C15H10O5) is an anthraquinone derivative, which has been identified in 17 families of natural plants (Zheng et al., 2021), including *Rheum palmatum*, *Polygonum cuspidatum*, *Polygonum multiflorum* (Ahn et al., 2016; Li et al., 2016), *Cassiae semen* (Yang et al., 2019), etc. These herbs have long been used as antibacterial, anti-inflammatory, anti-fibrotic and anticancer, anti-aging, anti-hyperlipidaemia, antidiabetic, neuroprotective, hepatoprotective, antioxidant, laxative and hypotensive activities and treatment of infection medicine in China (Peng et al., 2013; Lin L et al., 2015; Dong et al., 2017; Xiang et al., 2020). As a component of these medicinal materials, emodin also has the same medicinal effects for various diseases, including asthma, atopic dermatitis, osteoarthritis, diabetes and diabetic complications, atherosclerosis, Alzheimer’s disease, hepatic disease, constipation and several types of cancers and so on (Zheng et al., 2014; Dong et al., 2016). In recent years, there has been increasing evidence indicating that emodin has good antiviral properties and is commonly used in the treatment and prevention of epidemics caused by viruses.

Virus-infection diseases pose a significant treatment burden owing to their characteristics of recurrency and resulting complications. For example, seasonal influenza A virus (IAV) infection of patients with metabolic diseases may lead to acute lung injury (ALI) and acute respiratory distress syndrome (ARDS) (Bei et al., 2021), and HSV causes lifelong infections by establishing latency in neurons which promote recurrent disease and new infections when the immune system is weak (Ma et al., 2021). All of these features present difficulties for antiviral treatment, and we all know that the development of vaccines against many viruses is still in progress. Thus, there is a great need to explore novel drugs which can suppress different kinds of viruses and can antiviral infection *via* multiple perspectives. The efficacy of traditional Chinese medicine (TCM) in treating viral infectious diseases has been demonstrated in a number of public health events, including severe acute respiratory syndrome (SARS) in 2003 and coronavirus disease (COVID-19) in 2019. Similarly, herbal medicines and their active ingredients have been shown to have significant antiviral effects (Shao et al., 2021). There are some evidences (including in silico study and cell experiments) indicating that emodin might be effective therapy factor against SARS-CoV and COVID-19 (Ho et al., 2007; Schwarz et al., 2011; Batista et al., 2019; Basu et al., 2020; Caruso et al., 2020; Boozari and Hosseinzadeh, 2021; Nawrot-Hadzik et al., 2021; Rolta et al., 2021), suggesting that it may be an effective new antiviral agent. Through collection and collation of related literature, we found that emodin also has good antiviral effects on other viruses, so the aim of this review is to collect and present the current experimental evidence for the antivirus efficacy and underlying mechanisms of emodin, with a view to informing the development of new antiviral drugs.

## Biological Activity of Emodin

**FIGURE 1:**
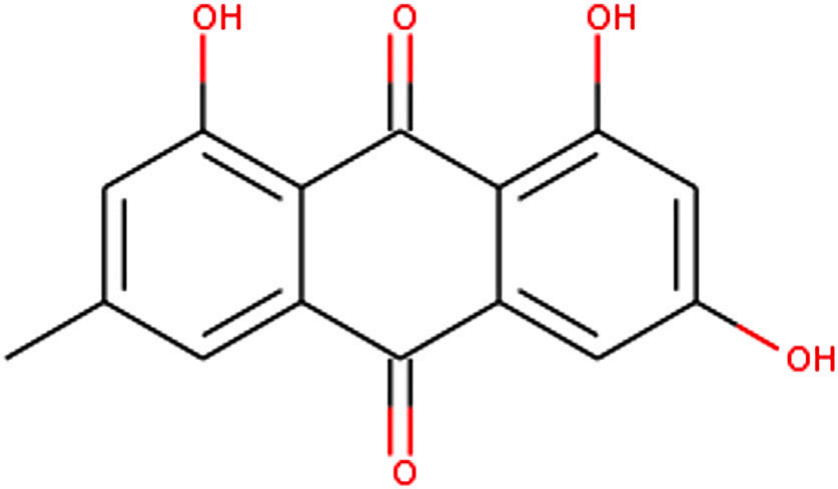
Chemical structure of emodin.

As an anthraquinone derivative, emodin’s basic chemical structure is an anthracene ring (tricyclic aromatic) with two ketone groups in position C9 and C10 (Monisha et al., 2016). The chemical structure of emodin is depicted in Figure 1. In general, emodin exerts its pharmacological activity at concentrations of a few tens of μM (Zheng et al., 2021). In pharmacokinetic experiments, after intragastric administration, emodin was quickly absorbed from the gastrointestinal tract and then rapidly metabolized to form its glucuronide, and the parent form of emodin was barely detectable *in vivo* (Shia et al., 2010). In rats’ experiment, biliary excretion of emodin reached a maximum at approximately 6 h; 70% of the biliary activity was in the form of conjugated emodin. Urinary excretion was 18 and 22% at 24 and 72 h, respectively, free emodin and emodin acid were principal metabolites found in the pooled urine (Mueller et al., 1999; Monisha et al., 2016). Although emodin is known to be rapidly soluble in DMSO, ethanol or alkaline solutions, it is practically insoluble in water (Zheng et al., 2021). In addition to this, the significant first-pass elimination effect of emodin in the liver and intestine determines its low oral bioavailability (Teng et al., 2012). In recent years, a numerous of studies aimed to overcome these shortcomings and much efforts have been achieved. To date, various methods have been investigated to enhance solubility of emodin, including physical or chemical modifications and the use of solubilisers or surfactants. A thermo-reversible gel based on poloxamer is a very attractive formulation for topical administration through the body surface to reduce metabolism and increase the solubility of emodin. Verified that thermoreversible poloxamer gel containing emodin indeed improved emodin solubility Ban et al. (2017). In addition, emodin-nicotinamide (EM-NCT) cocrystal form could improved emodin’s aqueous solubility, dissolution rate, and stability (Park et al., 2019). As for bioavailability, demonstrated that as a bioenhancer, piperine can enhance the bioavailability of emodin by inhibiting its glucuronidation Di et al. (2015). Furthermore, Two cocrystals of emodin (EM) with berberine chloride (BER), EM-BER 1) and 2 EM-BER-EtOH 2) has higher C-max and AUC compared with pure emodin, indicating higher bioavailability of that (Deng et al., 2018).

## Antiviral Activity of Emodin

### Anti-Herpes Simplex Virus Activities of Emodin

HSV including two subtypes, HSV-1 and HSV-2, which are large double-stranded DNA viruses of the Herpesviridae family and share 83% sequence homology in the protein coding region (Dolan et al., 1998). HSV infection is characterized by lifelong infection with intermittent clinical and subclinical viral reactivation, and it is the leading cause of genital ulcers around the world, named genital herpes (GH). Both HSV-1 and HSV-2 infection can cause GH, while HSV-2 is the main culprit, which infected 491.5 million people in 2016 worldwide (James et al., 2020). GH leading to great burden to individuals and to public health, since people infected with virus suffering painful, frequent genital lesions. On the other hand, HSV infection in early pregnancy can be transmitted through the placenta to the embryos, causing miscarriage, fetal malformations or permanent neurological damage, seriously affecting the quality of the birth population (Gupta et al., 2007). And the disruption of the mucosa caused by genital ulcers provides an entry point, thus facilitating HIV infection (Corey et al., 2004). At the same time, current treatment has not yet been able to completely eliminate latent HSV infection from the body, resulting in recurrence of the disease, which has a significant impact on the physical and mental health of patients and their quality of life. HSV-1 mainly causes cold sores on the lips, occasionally causes corneal lesions, and can also spread to the central nervous system causing serious diseases (Shukla and Valyi-Nagy, 2022). Antiviral drugs that target HSV viral DNA polymerase neither eradicate latent virus nor decrease the risk, frequency or severity of relapse. Meanwhile, vaccines against HSV are also still in progress and no breakthrough has been made. Therefore, it is essential to develop new antiviral drugs.

It has long been known that emodin shows antiviral effect on HSV-1 and HSV-2 (Table 1). In Vero cells, 10 μg/ml emodin were able to inactivate 5 × 10^7^ PFU/ml HSV-1 and 5 × 10^6^ PFU/ml HSV-2 (Andersen et al., 1991), and 2 μg/ml emodin could completely suppress 10^5^ PFU/ml HSV-1 (Cohen et al., 1996), and 21.5 ± 4.4 μM was sufficient to reduce 50% HSV-1 (30 PFU) virus yields without cytotoxic effect (Hsiang and Ho, 2008). While in HEp-2 (human laryngeal carcinoma) cells, emodin inhibited the replication of 100 TCID50/mL HSV-1 and HSV-2, with concentration of 50 μg/ml on HSV-1, and 25 μg/ml on HSV-2 (Xiong et al., 2011).

**TABLE 1:** Anti-HSV activities of emodin.

References	Cell lines/mice	Virus	Mechanism
Andersen et al. (1991)	Vero cell	HSV-1	Emodin inactivates 5 × 10^7^ PFU/ml HSV-1 and 5 × 10^6^ PFU/ml HSV-2
		HSV-2	
Cohen et al. (1996)	Vero cell	HSV-1	Emodin suppresses 105 PFU/ml HSV-1
Hsiang and Ho, (2008)	Vero cell	HSV-1	Emodin seduces 50% HSV-1 virus yields by inhibiting UL12 activity
Xiong et al. (2011)	HEp-2 cell	HSV-1	Emodin inhibits the replication of 100 TCID50/mL HSV-1 and HSV-2
		HSV-2	
Xiong et al. (2011)	BALB/c mice	HSV-1	Emodin shows good survival in HSV infected mice and clears HSV from brain, heart, liver and ganglia
		HSV-2	
Huang et al. (2021)	BALB/c mice	HSV-1	Emodin inhibits the inflammatory response in the brain of mice with herpes virus encephalitis by inhibiting TLR3 expression

In *in vivo* experiments, compared with the acyclovir-treated BALB/c mice, the 6.7 g/kg/day emodin group showed good survival in both HSV subtype-infected mice and longer mean time death (MTD) in HSV-1-infected mice. At the same time, emodin could clear HSV from brain, heart, liver and ganglia effectively (Xiong et al., 2011). 0.6 mg of emodin given to the HSV-1 infected male BALB/c mice daily for five consecutive days can effectively inhibit the inflammatory response in the brain of mice with herpesvirus encephalitis, the mRNA expressions of TLR3, TRIF, TRADD, TRAF6, traf3, p38, NEMO, and IRF3 are decreased, and the expressions of IL-6, TNF-α and IFN-β are decreased, indicating that emodin could inhibit the inflammatory response in the brain of mice with herpes virus encephalitis by inhibiting TLR3 expression (Huang et al., 2021).

The alkaline nuclease encoded by the UL12 gene of HSV-1 has endonuclease and exonuclease activities under alkaline pH conditions. Although UL12 is not required for either viral DNA synthesis or packaging, UL12 is required for efficiency of these processes. Therefore, HSV-1 UL12 can be a new target for anti-herpes virus drugs. C-Y Hsiang and T-Y Ho found that emodin could reduce the virus plaque formation in Vero cells by inhibiting UL12 activity, and the inhibitory effect may result from the interaction between emodin and critical catalytic amino acid residues of UL12 by docking analysis (Hsiang and Ho, 2008).

### Anti-Human Cytomegalovirus Activities of Emodin

HCMV is a very common herpes virus that infect a high percentage of the world’s population. After initial infection, HCMV is latent in the infected cells, thus causing lifelong infection, and usually without obvious clinical symptoms (Goodrum et al., 2012). However, HCMV infection (primary or (re)infection and reactivation) in immunocompromised individuals (e.g., HIV-infected persons, transplant recipients or children with congenital infection) can lead to serious complications (Deng et al., 2021). In addition, HCMV modulates the host immune response and promotes the modification of non-coding RNA and regulatory proteins, leading to an immunosuppressive tumor microenvironment. HCMV can also contribute to tumor survival by affecting cell proliferation and survival, invasion, immune evasion, immunosuppression and the production of angiogenic factors. Therefore, HCMV infection strongly associated with the development of tumors (El Baba and Herbein, 2021). Drug therapy currently approved for the treatment of systemic CMV infection has limited efficacy due to dose-limiting toxicity, and long-term treatment often results in drug resistance. Therefore, more effective and less toxic therapies are urgently needed to combat CMV infection (Alam et al., 2015). Current studies on the treatment of HCMV with emodin are all *in vitro* and the most commonly used cell line is human lung fibroblasts (MRC-5) cells (Table 2). Emodin showed antiviral activity against HCMV strain AD-169 (10^6.6^ PFU/ml) with 4.1 μM EC50 and 9.6 μM IC50. At the same time, emodin could effectively inhibit a ganciclovir resistant strain C8805-37 with 3.7 μM EC50 and 12.6 μM IC50 (Barnard et al., 1992). For strain AD169 with an MOI of 0.8, emodin could reduce the infectious yield with an EC50 of 4.9 μM (Alam et al., 2015). These evidences suggest that emodin has the potential to combat HCMV infection, but more validation and *in vivo* experiments are needed.

**TABLE 2:** Anti-HCMV activities of emodin.

References	Cell lines/mice	Virus	Mechanism
Barnard et al. (1992)	MRC-5 cells	strain AD-169	Emodin shows antiviral activity against strain AD-169 with 4.1 μM EC50 and 9.6 μM IC50 and inhibits strain C8805-37 with 3.7 μM EC50 and 12.6 μM IC50
		strain C8805-37	
Alam et al. (2015)	MRC-5 cells	strain AD169	Emodin reduces the infectious yield with an EC50 of 4.9 μM

### Anti-Epstein-Barr Virus Activities of Emodin

EBV is a gamma human herpesvirus that mainly infects B-cells and epithelial cells. EBV infection is the most common and persistent viral infection in humans, with approximately 95% of the world’s population remaining asymptomatic throughout their lives (Young et al., 2016). A small proportion of people infected with EBV present with infectious mononucleosis, which can cause persistent fatigue for up to 6 months and lead to serious neurological, hematological or hepatic complications. EBV was also the first human oncovirus to be identified and it is closely associated with several lymphomas and epithelial cancers especially in immunocompromised individuals, such as Burkitt’s lymphoma, Hodgkin’s lymphoma and nasopharyngeal carcinoma (NPC) (Cui and Snapper, 2021). In general, the utilizing of existing antiviral compounds is limited by toxic side effects, poor oral bioavailability and the risk of the emergence of resistant viral strains. There is a need to develop new drugs against EBV and virus-related diseases.

Replication of EBV plays an important role in the pathogenesis of NPC, and the relapse and metastasis in NPC patients remain major causes of mortality. Therefore, inhibition of EBV reactivation is now being considered as a goal for the therapy of NPC. As a natural product, emodin has the effect of anti-virus as well as anti-tumor. *In vitro* and *in vivo* studies have shown that emodin inhibits the expression of EBV lytic proteins Zta, Rta, EAD, and DNase and blocks virion production by repressing the transcription of EBV immediate early genes (Yiu et al., 2014; Wu et al., 2019). At the same time, emodin inhibits the tumorigenic properties induced by repeated EBV reactivation, including micronucleus formation, cell proliferation, migration, and matrigel invasiveness (Wu et al., 2019).

In addition, found that the cell proliferation of Burkitt’s lymphoma-derived Raji cells, which are EBV-positive cells, could be suppressed by the Polygonum cuspidatum ethyl acetate subfraction containing emodin (F3a) *via* increasing the intracellular reactive-oxygen species (ROS), activating the apoptosis-related proteins, and increasing the apoptosis percentage Yiu et al. (2021). These results mean that emodin may be a therapeutic drug for EBV-related tumors. Apart from this, EBV nuclear antigen EBNA1, a dimeric protein, which can bind to EBV genome sequences to initiate the process of DNA synthesis, is a potential therapeutic target for the treatment of EBV infection. Molecular docking revealed emodin bound to EBNA1 with high affinities, means that emodin may against HBV infection by inhibiting EBNA1 (Jakhmola et al., 2021). The above mechanisms are summarized in Table 3.

**TABLE 3:** Anti-EBV activities of emodin.

References	Cell lines/mice	Virus	Mechanism
Wu et al. (2019)	EBV-infected NPC cell lines NA and HA; SCID mice	EBV	Emodin inhibits the tumorigenic properties induced by repeated EBV reactivation, including micronucleus formation, cell proliferation, migration, and matrigel invasiveness
Yiu et al. (2014)	P3HR1 cells	EBV	Emodin inhibits the expression of EBV lytic proteins Zta, Rta, EAD, and Dnase, and blocks virion production by repressing the transcription of EBV immediate early genes
Yiu et al. (2021)	The Burkitt’s lymphoma-derived Raji cells	EBV	Polygonum cuspidatum ethyl acetate subfraction containing emodin (F3a) can increases the intracellular ROS, activats the apoptosis-related proteins, and increases the apoptosis percentage

### Anti-Coxsackievirus B Activities of Emodin

Coxsackieviruses are a group of envelope-free, orthotropic, single-stranded RNA viruses belonging to the small ribonucleic acid virus family of human enterovirus species (Simmonds et al., 2020). Coxsackieviruses are divided into group A (CVA) and group B (CVB). CVBs contain six virus types, CVB1-CVB6, which are common human pathogens associated with a wide range of diseases from gastrointestinal disorders to aseptic meningitis, myocarditis and pancreatitis, particularly in infants and children (Liu and Luo, 2021). Although the structure, molecular biology and associated pathophysiological mechanisms of CVB have been extensively studied, specific inhibitors have not yet been identified and applied to clinical studies. Therefore, research into natural or synthetic compounds is constantly ongoing in order to find suitable candidates for antiviral effectively (Hamdi et al., 2021). Several *in vitro* and *in vivo* studies showed anti-CVB activity of emodin (Table 4).

**TABLE 4:** Anti-CVB activities of emodin.

References	Cell lines/mice	Virus	Mechanism
Zhang H. M. et al. (2016)	HeLa cells	CVB3	Emodin inhibits viral replication through impairing translational machinery and suppression of viral translation elongation
	HL-1 cells		
	A/J mice		
Lin et al. (2019)	BALB/c mice	CVB3	Emodin decreases the HW/BW ratio, myocardial pathological score, the myocardial MDA level and serum cTnI and BNP levels, the TNF-alpha and IL-6 levels, and increase the myocardial SOD level of VMC mice
Zhang Y. F. et al. (2016)	BALB/c mice	CVB3	Emodin inhibits TLR4 and P38MAPK expression, thereby protecting cardiac tissue
Ding (2020)	mouse BV2 microglia	CVB3	Emodin inhibits the TLR3 pathway, decreasing levels of TLR3 protein, IL-6, NF-κB and IFN-β, as well as alleviates pathology of virus infected mice
	BALB/c mice		
Liu et al. (2013)	Hep-2 cells	CVB4	Emodin reduces CVB4 entry and replication on Hep-2 cells, increases survival rate, body weight and MTD of CVB4 infected mice, and decreases myocardial virus titers and pathologic scores/lesions, as well as inhibits CVB4-induced apoptosis *in vitro* and *in vivo*
	BALB/c mice		
Liu Z. et al. (2015)	HEp-2 cells	CVB5	Emodin could inhibit the replication of CVB5 by regulating cytokine (IFN-γ and TNF-α) expression

CVB3 is a primary causal agent of viral myocarditis (VMC). Zhang et al. explored the role and mechanism of anti-CVB3 of emodin through three cell lines and in A/J mice. CVB3 infected the immortalized human cardiomyocytes at an MOI of 20 for 24 h, the HeLa cells at an MOI of 10 for 5 h, and the HL-1 cells at an MOI of 10 for 14 h respectively and treated with 20 μM emodin at the same time. *In vivo*, 4-week-old male A/J mice were treated by 40 mg/kg emodin before 5 × 10^4^ pfu CVB3 infection, and the duration of administration lasts for 5 days. The results showed that emodin could inhibit CVB3 replication *in vitro* and in mice through multiple pathways of viral protein translation inhibition. Firstly, emodin suppressed translation initiation of ribosomal protein L32 *via* inhibiting Akt/mTOR (mammalian target of rapamycin) signaling and activating 4EBP1 (eukaryotic initiation factor 4R-binding protein 1). Secondly, emodin inhibited CVB3 VP1 (viral protein 1) synthesis by regulating Akt/mTORC1/p70S6K (p70 S6 kinase), ERK1/2 (extracellular signal-regulated kinase 1/2)/p90RSK (p90 ribosomal S6 kinase) and Ca^2+^/calmodulin. During this process, eEF2K is a major factor mediating cross-talk of signaling cascades which verified by inhibiting eEF2K with siRNA overexpression or inhibitor A484954. The above mechanisms ware also validated by overexpression and inhibition of Akt (Zhang HM et al., 2016). Apart from this, emodin could decrease the HW/BW ratio, myocardial pathological score, the myocardial MDA level and serum cTnI and BNP levels, TNF-α and IL-6 levels, and increase the myocardial SOD level of VMC mice (Lin et al., 2019), while attenuating cardiac injury in CVB3-infected BALB/c mice by inhibiting TLR4 and P38MAPK expression, thereby protecting cardiac tissue (Zhang YF et al., 2016).

In addition to myocarditis, CVB3 is also the main culprit in Hand, Foot and Mouth Disease (HFMD), and a high prevalence of CVB3 has been repeatedly found in Chinese patients with HFMD (Ding et al., 2020). Meanwhile, encephalitis in HFMD is a serious threat to children’s health and life. Ding et al. explored the effect of emodin on CVB3 infection caused HFMD using mouse BV2 microglia as a cellular model. The results suggested that emodin could inhibit the TLR3 pathway which can recognize virus and initiate innate immune responses to suppress viral infection. Then using three-week-old male BALB/c mice to establish animal model by injecting 20 µl of virus solution directly into the brain, then 80 mg/kg, 40 mg/kg and 20 mg/kg emodin daily intragastrical for 7 days. Emodin displayed notable effects on alleviating pathology, decreasing TLR3 protein in brain tissues and expression levels of IL-6, NF-κB, IFN-β in serum, and the 80 mg/kg emodin group has the best effect of anti-inflammatory (Ding et al., 2020), which suggested emodin may be effective agent to HFMD.

Besides CVB3, emodin also exhibits anti-viral effect to CVB4. CVB4 can cause a broad range of diseases, including myocarditis, pancreatitis, hepatitis, meningoencephalitis, gastroenteritis, necrotizing enterocolitis, pneumonia and even death in neonates. Emodin can reduce CVB4 entry and replication on Hep-2 cells in a concentration- and time-dependent manner, and the 4–6-week BALB/c mice orally treated with different dosages of emodin displayed a dose dependent increase of survival rate, body weight and prolonged MTD, accompanied by significantly decreased myocardial virus titers and pathologic scores/lesions. Moreover, emodin could inhibit CVB4-induced apoptosis *in vitro* and *in vivo* which represents the cardio protection of emodin (Liu et al., 2013).

Coxsackievirus B5 (CVB5) is one of the five most common types of enterovirus (EV) and is associated with encephalitis and myocarditis in immunocompromised children and central nervous system disease in the elderly (Zhong et al., 2009). Therefore, the search for evidence and mechanisms of emodin against CVB5 infection is also warranted. Liu et al. found that emodin had potent inhibitory activities against 100 TCID50/ml CVB5 in HEp-2 cells, with the 50% effective concentration (EC50) ranging from 13.06 to 14.27 μmol/L. It acted as a biological synthesis inhibitor against CVB5 in a concentration- and time-dependent manner. Moreover, emodin could decrease the mRNA expression of IFN-α but enhance TNF-γ expression significantly compared to the model group, suggesting that emodin could inhibit the replication of CVB5 by regulating cytokine (IFN-γ and TNF-α) expression (Liu Z et al., 2015).

### Anti-Hepatitis B Virus Activities of Emodin

Chronic HBV infection is a major global health problem and an important cause of complications, including liver failure, development of liver cirrhosis (LC) and hepatocellular carcinoma (HCC) (Liaw and Chu, 2009). There are approximately two billion people worldwide infected by HBV, resulting in approximately one million deaths each year (Polaris Observatory Collaborators, 2018; Chien and Liaw, 2022). The population of infection with HBV is still increasing even though vaccination can prevent HBV, and the currently recognized effective antiviral treatment drugs have disadvantages such as high adverse effects and high prices. In recent years, researchers have been investigated the antiviral activity of various products from plants in order to find effective alternative medicines (Chai et al., 2019).

Emodin may be a new treatment for HBV infection (Table 5). The inhibitory effect of emodin on HBV DNA replication and HBsAg secretion is time- and concentration-dependent *in vitro*. The human hepatoma G2.2.15 (HepG2.2.15) cell line stably expresses HBV particles. After exposure to three different concentrations of emodin (12.5 mg/L, 25 mg/L and 50 mg/L) for 3, 6, and 9 days in HepG2.2.15 cells, the inhibition rates of extracellular HBV DNA, HBsAg, and HBeAg were significantly increased. And the inhibition rates increased as time passed and peaked after 9 days of treatment, and 50 mg/L emodin treatment exhibited the best antiviral effect (Dang et al., 2006). In addition, in HBV transgenic mice, 57.59 mg/kg/d emodin and 287.95 mg/kg/d astragalus polysaccharide (APS) Co-treatment for 3 weeks could significantly decrease HBV DNA levels, the contents of HBsAg, HBeAg and HBcAg when compared with control group, which means emodin may function as a complementary factor in the treatment of HBV infection (Dang et al., 2009).

**TABLE 5:** Anti-HBV activities of emodin.

References	Cell lines/mice	Virus	Mechanism
Dang et al. (2006)	HepG2.2.15 cells	HBV	Emodin inhibits the extracellular HBV DNA, HBsAg and HBeAg, and the inhibition efficacy increases as time passed and peaked after 9 days of treatment
Dang et al. (2009)	HBV transgenic mice	HBV	Emodin and APS Co-treatment could decrease HBV DNA levels, the contents of HBsAg, HBeAg and HBcAg

The HBV core protein contains 183 residues that self-assemble to form the viral capsid. In infected cells, the HBV core protein regulates nearly every step of the viral replication process. It is an excellent target for the development of novel, virus-selective and effective antiviral drugs to improve treatment options for HBV infectious diseases. Emodin derivatives showed promising inhibitory characteristics to orientation of capsid assembly by core proteins using molecular docking and dynamic simulation, indicating that viral replication would be inhibited by emodin derivatives (Firdayani et al., 2018).

### Anti-Influenza A Virus Activities of Emodin

IAV belongs to one of the three influenza genera (including A, B, and C) of the family Orthomyxoviridae and is a segmented, negative-sense ribonucleic acid virus (Atkin-Smith et al., 2018). Influenza infections have a serious impact on health worldwide, causing almost 3–5 million cases of critical illness and approximately 250,000–500,000 deaths worldwide each year (Gui and Chen, 2021). Currently, the classical antiviral drugs (amantadine, ribavirin or oseltamivir) are widely used in the clinic. Nevertheless, new effective drugs are still needed because of potential toxicities, rapid emergence of antiviral resistance and high prices of existing drugs (Zhi et al., 2019).

Emodin is a highly promising anti-IVA agent (Table 6). The current studies on emodin for IAV are based on A549 lung cancer cells, and A549 cells are the most common used cell type of researching anti-IVA effect of emodin. For IAV (PR8), the mechanism of the anti-viral effect of emodin in A549 cells was to activate PPARα/γ and AMPK, decrease fatty acid biosynthesis, and increase intracellular ATP levels. In order to further prove that PPARα/γ and AMPK are the key proteins that inhibit PR8 replication, inhibitors were used, and it was found that the inhibitors of PPARα/γ and AMPK weakened the antiviral effect of emodin (Bei et al., 2021). For H1N1, inhibiting the expression of hemagglutinin and neuraminidase, increasing the expression of interferon beta (IFN-β) through Toll-like receptor 9 (TLR9) are the key ways to inhibit the replication of it (Lin CJ et al., 2015). In *vivo* studies, emodin also significantly protected mice from IAV infection and pneumonia (Bei et al., 2021). Meanwhile, though experiments on A549 cells and C57BL/6J mice, DAI et al. comprehensively explored the mechanism of emodin’s antiviral effect to H1N1. The results showed that emodin could significantly inhibit IAV (ST169, H1N1) replication, reduce the expressions of TLR2/3/4/7, MyD88 and TRAF6, decrease phosphorylation of p38/JNK MAPK and nuclear translocation of NF-kB p65, those are crucial to H1N1 infection and replication. Nrf2 signaling pathway is a classic anti-inflammatory pathway, and activation of which can inhibit the activation of TLR pathways. Suppression of Nrf2 *via* siRNA markedly blocked the inhibitory effects of emodin on TLR4, p38/JNK, and NF-kB pathways and on IAV-induced production of IL-1, IL-6 and expression of IAV M2 protein. Meanwhile, Nrf2 signaling pathway is also essential to anti-oxidate. Therefore, emodin could activate the Nrf2 pathway and decreased ROS levels, increased GSH levels and GSH/GSSG ratio, and upregulated the activities of SOD, GR, CAT and GSH-Px. Similarly, Dai et al. also clarified that emodin has a therapeutic effect on H1N1infected acute lung injury (ALI) mice. Emodin increased the survival rate of mice, reduced lung edema, pulmonary viral titer and inflammatory cytokines (IL-1β, IL-6, IL-8, TNF-a), and improved lung histopathological changes (Dai et al., 2017).

**TABLE 6:** Anti-IVA activities of emodin.

References	Cell lines/mice	Virus	Mechanism
Bei et al. (2021)	A549 cells	IAV (PR8)	Emodin activates PPARα/γ and AMPK, decrease fatty acid biosynthesis, and increases intracellular ATP levels. Emodin also protects mice from IAV infection and pneumonia
	BALB/c mice		
Lin et al. (2019)	A549 cells	H1N1	Emodin inhibits IVA replication *via* regulating TLR-9-induced IFN-β production
Dai et al. (2017)	A549 cells and C57BL/6J mice	H1N1	Emodin can inhibit IAV replication and influenza viral pneumonia by activating Nrf2 signaling and inhibiting TLR4, p38/JNK MAPK and NF-κB pathways

### Anti-SARS-CoV Activities of Emodin

Coronaviruses belong to the family of Coronaviridae (subfamily Coronaviridae), whose members infect a wide range of hosts, resulting symptoms and illnesses ranging from the common cold to severe and ultimately fatal diseases such as severe acute respiratory syndrome coronavirus (SARS-CoV), Middle East respiratory syndrome coronavirus (MERS-CoV), and currently coronavirus disease 2019 (COVID-19) (Dhama et al., 2020). These diseases pose a great threat to people’s lives and health and has a huge impact on the global economy. To date, few measures are available to effectively treat COVID-19. Therefore, there is an urgent need for new drugs to combat the disease or for new treatments for these kinds of extremely dangerous coronavirus diseases (Yu et al., 2021).

In 2003, SARS resulted in progressive respiratory failure and death in close to 10% of infected individuals (Ksiazek et al., 2003), and studies showed that emodin exhibits anti-SARS-CoV potential (Table 7). SARS-CoV S protein is a large type I membrane glycoprotein projection from viral envelope, mutations in this gene dramatically affect the virulence, pathogenesis, and host cell tropism. While angiotensin-converting enzyme 2 (ACE2) was identified as a functional receptor for SARS-CoV (W. Li et al., 2003), suggesting that blocking the binding of the S protein with its cellular receptor can prevent virus entry. Recombinant SARS-CoV S protein and S protein pseudotyped retrovirus was used to explore the interaction between S protein and ACE2, it was found that S protein binds to ACE2 in a dose-dependent manner, while emodin blocked the binding of S protein to ACE2 in a dose dependent manner, with IC_50_ value of 200 μM. At the same time, emodin can also inhibit the infectivity of S protein-pseudotyped retrovirus to ACE2-expression Vero E6 cells. The above data suggest that emodin may be considered as a potential lead therapeutic agent in the treatment of SARS (Ho et al., 2007). Apart from this, SARS-CoV has an open reading frame, ORF-3a, that encodes an ion-osmotic channel in infected cells, and the activity of the 3a protein may affect viral release. Rhabdomyosarcoma cells (RD cells) were used to explore the effect of emodin on viral replication *via* the 3a protein. It was found that 100 μM emodin could inhibit the 3a ion channel of the coronaviruses SARS-CoV and HCoV-OC43 and the viral release of HCoV-OC43, with a K1/2 value of about 20 μM (Schwarz et al., 2011). These data demonstrated that S protein, ACE2 and ORF-3a may be the effective targets of emodin anti- SARS-CoV.

**TABLE 7:** Anti-SARS-CoV activities of emodin.

References	Cell lines/mice	Virus	Mechanism
Ho et al. (2007)	Vero E6 cells	SARS-CoV	Emodin blocks the S protein and ACE2 interaction in a dose-dependent manner and inhibits the infectivity of S protein-pseudotyped retrovirus
Schwarz et al. (2011)	Rhabdomyosarcoma cells	SARS-CoV	Emodin inhibits the 3a ion channel
Nawort-Hadzik et al. (2021)	/	SARS-CoV-2	Emodin reveals over 50% inhibition of SARS-CoV-2 Mpro

17 years later, in December 2019, a novel coronavirus, defined by the World Health Organization (WHO) as severe acute respiratory syndrome coronavirus 2 (SARS-CoV-2) in January 2020, re-emerged, known as COVID-19. The main protease (Mpro, also called 3CLpro) is the nonstructural proteins of the virus, and inhibition of this enzyme could prevent the replication of the virus. Found that emodin revealed over 50% inhibition of SARS-CoV-2 Mpro, suggesting the beneficial effects of emodin on COVID-19 Nawrot-Hadzik et al. (2021). In order to improve the activity of emodin against human coronavirus NL63 (HCoV-NL63) and also to generate a set of initial SAR guidelines, Monika et al. prepared emodin derivatives and found that halogenation of emodin can improve the antiviral activity of which. Moreover, the most active compound was the iodinated emodin analogue E_3I, the anti-HCoV-NL63 activity of which was comparable to that of remdesivir (Horvat et al., 2021). Of course, studies on emodin against COVID-19 to date are all based on *in vitro* experiments and there is limited discussion of its mechanisms, so more researches are needed to demonstrate the important role of emodin could play in this plague.

### Other Viruses

In addition to the inhibitory effects of the common viral infections mentioned above, there is evidence that emodin may also exert antiviral effects against other viruses (Table 8). VHSV is a negative RNA enveloped virus. Emodin exhibited anti-VHSV-07.71 effect in epithelioma papulosum cyprini (EPC) cell cultures (Alves et al., 2004). Human EV71 is the causative agent for HFMD outbreaking in Asia. Emodin (29.6 μmol/L) effectively inhibited viral replication thus protecting MRC5 cells from EV71-induced cytopathic effects by inhibiting viral maturation and diminishing cell cycle arrest at S phase (Zhong et al., 2017). Apart from that, emodin exhibited significant prophylactic effects against DENV-2 (two doses, 45 and 90 PFU) infectivity treating before infection (Batool et al., 2018) and reduced the infectivity of ZIKV approximately 83.3% (from 7.8 × 10^3^ PFU/ml to 1.3 × 10^3^ PFU/ml) in Vero E6 cells (Batista et al., 2019).

**TABLE 8:** Other virus.

References	Cell lines/mice	Virus	Mechanism
Alves et al. (2004)	epithelioma papulosum cyprini (EPC) cell	VHSV	Emodin exhibits anti-VHSV effect in epithelioma papulosum cyprini (EPC) cells
Zhong et al. (2017)	MRC5 cells	EV71	Emodin inhibits viral replication by inhibiting viral maturation and diminishing cell cycle arrest at S phase
Batool et al. (2018)	Baby hamster kidney (BHK-21) cells	DENV-2	Emodin exhibits prophylactic effects against dengue virus serotype 2 (DENV-2) infectivity treated before infection
Batista et al. (2019)	Vero E6 cells	ZIKV	Emodin reduces the infectivity of ZIKV approximately 83.3%

## Toxicity of Emodin

Despite the wide range of pharmacological effects of emodin, we cannot ignore its side effects, especially the toxicity on organs. The liver is one of the main target organs in drug toxicology. Intracellular metabolomic analysis showed that emodin significantly disrupted cellular glutathione and fatty acid metabolism, and the level of emodin-cysteine adduct increased with increasing emodin concentrations, which suggest a cytotoxic effect of emodin on the metabolic pathways of human hepatocytes (Liu X et al., 2015). Meanwhile, Wang et al. investigated the nephrotoxicity of emodin by inducing HK-2 cells (a human proximal tubular epithelial cell line) apoptosis *via* mitochondrial pathway (Wang et al., 2015). In addition to this, emodin has been reported to be reproductively toxic and genotoxic (Li et al., 2010; Oshida et al., 2011; Luo et al., 2015). At the same time, anthraquinones (AQ) such as emodin and chrysophanol exerts the chronic effects on organ weights and over 30 haematological, biochemical and histological parameters, and irreversible pathological changes can be caused at very high doses (4000 mg/kg) (Islam et al., 2015). Therefore, long-term high doses administration should be completely avoided during pregnancy, and it is essential to improve bioavailability and thus reduce the dose administered to achieve some reduction in drug toxicity.

## Conclusion

Viral infections are characterized by variables and the possibility of lifelong latency, and easy to develop resistance to antiviral drugs. In addition to inhibiting viral replication itself, the treatment of various related diseases caused by virus is also a major challenge. As a common natural product, emodin and its antiviral efficacy and mechanism have been explored and studied for about 30 years. The current researches on emodin are all based on cells and mice, and the viruses that can be suppressed by emodin including HSV-1, HSV-2, HCMV, HBV, CVB (type 3–5), EBV, IVA, SARS-CoV, VHSV, EV71, DENV-2, and ZIKV. The specific anti-virus mechanisms of emodin involved vary from virus to virus, while the common denominator is the ability to suppress the inflammatory response caused by viral infection, such as decreasing the expression of IL-6, TNF-α and IFN-β. Emodin not only exhibits the effect of inhibiting replication and infection of various viruses, but also has a certain recovery effect on tissue damage caused by virus infection. And some of its effects are even comparable to that of the commonly used antiviral drug acyclovir.

The current evidence is enough to show that emodin is a promising antiviral drug, but it is undeniable that more research is needed in the future to explain its antiviral mechanism, as well as human experiments to verify its safety and efficacy and possibility of treating viral infectious diseases as a single agent or in combination with other drugs.

## Abbreviations

HSV-1, herpes simplex virus type 1; HSV-2, herpes simplex virus type 2; HCMV, human cytomegalovirus; EBV, Epstein-Barr virus; CVB, coxsackievirus B; HBV, hepatitis B virus; IAV, influenza A virus; VHSV, viral haemorrhagic septicaemia rhabdovirus; EV71, enterovirus 71; DENV-2, dengue virus serotype 2; ZIKV, Zika virus; ARDS, acute respiratory distress syndrome; SARS, severe acute respiratory syndrome; GH, genital herpes; MTD, mean time death; NPC, nasopharyngeal carcinoma; ROS, reactive-oxygen species; VMC, viral myocarditis; HFMD, Hand, Foot and Mouth Disease; EC50, 50% effective concentration; TLR, Toll-like receptor.
